# Editorial: Emerging targeted and immunotherapeutic strategies in oncology: from solid tumors to hematologic malignancies

**DOI:** 10.3389/fphar.2026.1965471

**Published:** 2026-09-04

**Authors:** Ximena Maria Muresan, Adrian Bogdan Tigu, Andrei Roman, Arianna Romani

**Affiliations:** 1 Department of Translational Medicine and Rare Diseases, Institute for Biomedical Research – MEDFUTURE, University of Medicine and Pharmacy “Iuliu Hațieganu”, Cluj-Napoca, Romania; 2 University of Medicine and Pharmacy “Iuliu Hațieganu”, Cluj-Napoca, Romania; 3 Department of Translational Medicine and LTTA Centre, University of Ferrara, Ferrara, Italy

**Keywords:** drug resistance, hematologic malignancies, immunotherapy, precision oncology, small molecules, solid tumors, targeted therapy

## Introduction

Cancer remains among the leading causes of death worldwide, with an estimated 20 million new cases and nearly 10 million deaths in 2022 and a burden projected to grow substantially over the coming decades (Bray et al., 2024). The conceptual framework of cancer as a disease of dysregulated hallmark capabilities, sustained proliferation, evasion of growth suppression and cell death, immune escape, and genome instability, among others, has reframed these capabilities as actionable therapeutic targets (Hanahan, 2022). Over the past two decades, oncology has accordingly shifted from cytotoxic chemotherapy toward agents designed to engage specific molecular lesions (Segota and Bukowski, 2004), and toward a precision-medicine paradigm in which tumor genotype, including rare driver mutations, guides treatment selection (Nussinov et al., 2019).

Immunotherapy has been the most consequential addition to this arsenal. Immune checkpoint inhibitors targeting the PD-1/PD-L1 and CTLA-4 axes have produced durable responses across many tumor types, although only a subset of patients benefit and predictive biomarkers remain imperfect (Arafat Hossain, 2024; Affolter et al., 2022). In parallel, adoptive cell therapies have transformed the treatment of hematologic malignancies: chimeric antigen receptor (CAR) T cells achieve deep remissions in B-cell leukemias and lymphomas but face challenges of toxicity, antigen escape, and limited activity in solid tumors (Sharma et al., 2023), motivating alternative effectors such as engineered natural killer cells with a more favorable safety profile (Berrien-Elliott et al., 2015). The therapeutic toolkit has broadened further to include engineered biologics and targeted delivery platforms, aptamer-based agents (Zeng et al., 2025) and antibody-drug conjugates (Sun et al., 2023) that couple molecular specificity to potent cytotoxic payloads.

Yet resistance remains the defining obstacle of targeted and immune-based therapy. Acquired resistance to kinase inhibitors, exemplified by third-generation EGFR inhibitors (He et al., 2021), and intrinsic or evolving resistance to cytotoxic backbones such as platinum agents (Yang et al., 2022) and antibody-drug conjugates (Li et al., 2024) repeatedly blunt initial responses. Much of this resistance converges on a limited set of signaling and epigenetic nodes. The PI3K/Akt/mTOR axis integrates survival and proliferation signals and is among the most frequently dysregulated pathways in human cancer (Chamcheu et al., 2019), while chromatin readers and writers, notably the bromodomain protein BRD4 (Donati et al., 2018; Shu et al., 2020) sustain oncogenic transcriptional programs. These nodes have inspired new modalities, including targeted protein degraders (PROTACs) that eliminate rather than merely inhibit their targets (Li and Song, 2020) and strategies aimed at otherwise “undruggable” transcription factors (Du et al., 2024).

Hematologic malignancies have been proving ground for many of these advances, from risk-adapted regimens in acute lymphoblastic leukemia (Inaba and Mullighan, 2020) and tyrosine kinase inhibitors that have made treatment-free remission a realistic goal in chronic myeloid leukemia (Rinaldi and Winston, 2023), to menin inhibition in KMT2A-rearranged and NPM1-mutated acute myeloid leukemia (Bi and Zhou, 2025). Progress across both solid and liquid tumors increasingly depends on the tools used to discover and evaluate therapies: physiologically faithful three-dimensional culture and bioprinted models for preclinical testing (Tian et al., 2026), molecularly defined patient selection as in ALK-rearranged lung cancer (Wang and Wang, 2020) and immune-based treatment of gastric cancer (Zhang et al., 2024), and real-world evidence to confirm that trial findings translate into everyday practice (Dodkins et al., 2023). It is at this intersection of new targets, new modalities, faithful models, and rigorous real-world evaluation that the present Research Topic is positioned.

## The Research Topic at a glance

The past decade has redrawn the map of cancer therapeutics. The blunt cytotoxicity of conventional chemotherapy is steadily giving way to agents that engage defined molecular vulnerabilities and to strategies that recruit the patient’s own immune system against the tumor. Yet the promise of precision and immune-based oncology is tempered by persistent obstacles: intrinsic and acquired resistance, immune evasion, narrow therapeutic windows, immune-mediated toxicity, and the gap between controlled trials and everyday clinical practice. This Research Topic, “*Emerging Targeted and Immunotherapeutic Strategies in Oncology: From Solid Tumors to Hematologic Malignancies*,” was conceived to capture how investigators are confronting these obstacles across the full arc of translation, from target discovery and preclinical modeling to real-world outcomes and pharmacoeconomics.

The Research Topic brings together 21 articles, 11 original research papers, 6 case reports, 3 reviews, and 1 systematic review with meta-analysis, contributed by research groups across Europe, Asia, and the Middle East and published in Frontiers in Pharmacology, Oncology, Immunology, and Medicine. Together they span the disease spectrum promised in the title, from epithelial solid tumors to aggressive leukemias and lymphomas, and they cut across the therapeutic modalities that define contemporary oncology: small molecules, epigenetic agents, cell-based therapies, bispecific and checkpoint antibodies, and engineered biologics. Rather than summarize each contribution in isolation, we group them into the themes that emerged organically from the submissions.

## Small-molecule and epigenetic targeting of solid tumors

Several contributions tackle targets long considered difficult to drug. Liao et al. addressed the MYC-driven survival circuitry of gastric cancer indirectly, exploiting the bromodomain protein BRD4 as a co-activator dependency; combining transcriptomic analysis of TCGA and GEO cohorts with CRISPR-Cas9 deletion and PROTAC-mediated degradation (ARV-771), they nominate BRD4 as a tractable vulnerability where MYC itself is not. Zhang and Lou review the mitochondrial dimension of prostate cancer, oxidative phosphorylation dependence, mtDNA mutation, and Bcl-2–family apoptotic balance, as a source of emerging therapeutic targets. These works illustrate a recurring strategy in the Research Topic: when a canonical oncoprotein resists direct inhibition, its supporting infrastructure becomes the target.

## Novel modalities and physiologically relevant preclinical models

A second thread pushes beyond conventional small molecules toward engineered biologics and more faithful model systems. Vasilescu et al. engineered a double-stranded DNA minicircle to act as a decoy that sequesters STAT3 in SKOV3 ovarian cancer cells, while a companion study from the same group (Vasilescu et al.) fused an anti-B7-H3 affibody to the membranolytic peptide magainin-2 to create a cytotoxic biologic against acute myeloid leukemia. Bompan et al. evaluated the MDM2 inhibitor nutlin-3a in p53 wild-type retinoblastoma using both 2D cultures and 3D-bioprinted models, directly answering this Research Topic’s call for spheroid, organoid, and bioprinted systems that better recapitulate tumor biology. Collectively these studies show target validation increasingly paired with delivery innovation and model realism.

## Engineering the immune response

Immune engineering features prominently. Cianga et al. provide a synthesis of chimeric antigen receptor natural killer (CAR-NK) cell therapy, proposing an “evasion-to-solution” framework that maps specific tumor immune-escape mechanisms, antigen loss, lineage plasticity, epitope masking, trogocytosis, onto corresponding next-generation engineering solutions such as dual-antigen and low-affinity receptors, iPSC-derived platforms, and multiplex gene editing. The anti-B7-H3 affibody study (Vasilescu et al.) complements this by targeting an immune-checkpoint ligand associated with acquired resistance. These contributions reflect a maturing view of immunotherapy in which the tumor’s escape routes are anticipated and engineered against, rather than discovered after failure.

## Hematologic malignancies and the transplant setting

The field of hematology encompasses a variety of topics, particularly the incorporation of sequencing and established methods. Antohe et al. provide real-world data on the use of blinatumomab and inotuzumab for the treatment of adult patients with relapsed/refractory B-AL, while additionally demonstrating how disease burden and relapse timing can be leveraged in the selection of treatment agents. Bica et al. performed an assessment of donor lymphocyte infusion + azacitidine following allogeneic transplantation in pediatric AML and Wang et al. presented evidence-based recommendations for the individualized dose of anti-thymocyte globulin that will effectively prevent graft-versus-host disease while maintaining a good response rate. Huang et al. successfully proved the effectiveness of drug combinations between everolimus and gemcitabine in relapsed/refractory peripheral T-cell lymphoma. Overall, the key point presented in these works is that the combination of established drugs is the only way to achieve real success in the field.

## Overcoming and understanding therapeutic resistance

Resistance, the central adversary of targeted therapy, is confronted directly. Hakim et al. identified 3-mercaptopyruvate sulfurtransferase–derived hydrogen sulfide as a driver of chemoresistance in triple-negative breast cancer acting through PI3K/Akt/mTOR signaling, nominating this gasotransmitter pathway as a chemosensitizing target. In the clinic, Zhang et al. reported that the dual mTORC1/2 inhibitor onatasertib re-sensitized an immunotherapy-refractory nasopharyngeal carcinoma to PD-1 blockade, and Chandra et al. described the first successful rechallenge of BRAF/MEK inhibition, enabled by ruxolitinib, after a hemophagocytic-lymphohistiocytosis–like hyperinflammatory syndrome in BRAF V600E lung adenocarcinoma, a reminder that managing toxicity can be as decisive as managing the tumor.

## Precision oncology in rare tumors and diagnostic challenges

Case reports in this Research Topic do more than document rarity; they extend the molecular reach of precision oncology into settings where evidence is scarce. Liu et al. reported the first case of FGFR1 rearrangement in KIT-negative mast cell leukemia, redirecting therapy to an FGFR1 inhibitor. Zaro et al. achieved durable disease stability with pazopanib in disseminated hepatic epithelioid hemangioendothelioma, and Lang and Xiong explored radiotherapy followed by anlotinib and toripalimab in relapsed low-grade myofibroblastic sarcoma. Zhao et al. described ALK-rearranged lung adenocarcinoma masquerading as anterior uveitis, with intraocular and intracranial metastases resolving on alectinib, underscoring how molecular diagnosis reframes even the most atypical presentations.

## Real-world evidence, pharmacoeconomics, and the research landscape

Finally, a cluster of studies bridges controlled evidence and clinical reality. Micu et al. analyzed 201 chronic myeloid leukemia patients managed over more than two decades in Romania, using a large-language-model pipeline to structure unstructured discharge records and machine learning to predict treatment-free-remission eligibility, a pointed example of how computational tools can unlock real-world data from under-represented regions. Cai et al. synthesized the efficacy and safety of tebentafusp in metastatic uveal melanoma across 850 patients in a systematic review and single-arm meta-analysis. Xiang et al. evaluated the cost-effectiveness of the PD-1/CTLA-4 bispecific cadonilimab in HER2-negative gastric cancer, and Liu et al. mapped a decade of research on ALK-tyrosine kinase inhibitors in non-small cell lung cancer bibliometrically. Together they insist that a therapy’s value is measured not only by response rates but by cost, accessibility, and reproducibility outside the trial.

## Concluding remarks

Read together, these 21 contributions trace a coherent narrative. Target discovery is moving toward previously “undruggable” nodes and their supporting networks; therapeutic modalities are diversifying from small molecules to engineered cells, decoys, and bispecific antibodies; preclinical models are becoming more physiologically faithful; and evaluation is extending beyond efficacy to encompass resistance mechanisms, toxicity management, cost, and real-world reproducibility. Recurring motifs, the mTOR axis appearing in three distinct settings, the strategic combination of established agents, and the recurrent theme of anticipating rather than reacting to resistance, suggest that the field’s next advances will come as much from how we deploy and sequence therapies as from the discovery of new ones.

We thank all authors for their rigorous and diverse contributions, the reviewers whose scrutiny strengthened every paper, and the Frontiers editorial staff for their support. We hope this Research Topic serves as both a snapshot of a rapidly moving field and a stimulus for the translational, multidisciplinary work that patients with solid tumors and hematologic malignancies urgently need.
